# A new glaucoma hypothesis: a role of glymphatic system dysfunction

**DOI:** 10.1186/s12987-015-0012-z

**Published:** 2015-06-29

**Authors:** Peter Wostyn, Debby Van Dam, Kurt Audenaert, Hanspeter Esriel Killer, Peter Paul De Deyn, Veva De Groot

**Affiliations:** Department of Psychiatry, PC Sint-Amandus, Reigerlostraat 10, 8730 Beernem, Belgium; Department of Biomedical Sciences, Laboratory of Neurochemistry and Behavior, Institute Born-Bunge, University of Antwerp, Universiteitsplein 1, 2610 Antwerp, Belgium; Department of Psychiatry, Ghent University Hospital, De Pintelaan 185, 9000 Ghent, Belgium; Department of Ophthalmology, Kantonsspital Aarau, Buchserstrasse, Aarau, 5001 Switzerland; Department of Neurology and Memory Clinic, Middelheim General Hospital (ZNA), Lindendreef 1, 2020 Antwerp, Belgium; Department of Neurology and Alzheimer Research Center, University of Groningen and University Medical Center Groningen, Hanzeplein 1, 9700 RB Groningen, The Netherlands; Department of Ophthalmology, Antwerp University Hospital, Wilrijkstraat 10, 2650 Antwerp, Belgium

**Keywords:** Cerebrospinal fluid circulation, Cerebrospinal fluid clearance, Glymphatic pathway, Glaucoma, Alzheimer’s disease, Intracranial pressure

## Abstract

In a recent review article titled “A new look at cerebrospinal fluid circulation”, Brinker et al. comprehensively described novel insights from molecular and cellular biology as well as neuroimaging research, which indicate that cerebrospinal fluid (CSF) physiology is much more complex than previously believed. The glymphatic system is a recently defined brain-wide paravascular pathway for CSF and interstitial fluid exchange that facilitates efficient clearance of interstitial solutes, including amyloid-β, from the brain. Although further studies are needed to substantiate the functional significance of the glymphatic concept, one implication is that glymphatic pathway dysfunction may contribute to the deficient amyloid-β clearance in Alzheimer’s disease. In this paper, we review several lines of evidence suggesting that the glymphatic system may also have potential clinical relevance for the understanding of glaucoma. As a clinically acceptable MRI-based approach to evaluate glymphatic pathway function in humans has recently been developed, a unique opportunity now exists to investigate whether suppression of the glymphatic system contributes to the development of glaucoma. The observation of a dysfunctional glymphatic system in patients with glaucoma would provide support for the hypothesis recently proposed by our group that CSF circulatory dysfunction may play a contributory role in the pathogenesis of glaucomatous damage. This would suggest a new hypothesis for glaucoma, which, just like Alzheimer’s disease, might be considered then as an imbalance between production and clearance of neurotoxins, including amyloid-β.

## Background

In a recent review article titled “A new look at cerebrospinal fluid circulation” published in *Fluids and Barriers of the CNS*, Brinker et al. [1] comprehensively described novel insights from molecular and cellular biology as well as neuroimaging research, which indicate that cerebrospinal fluid (CSF) physiology is much more complex than previously believed. According to the traditional understanding of CSF physiology, the majority of CSF is produced by the choroid plexus, circulates through the ventricles, the cisterns, and the subarachnoid space (SAS) ultimately to be absorbed into the venous blood system across arachnoid villi and/or via perineural spaces of the cranial nerves into the cervical lymphatics [1]. Recently, the classic CSF circulation theory has been increasingly challenged and a new hypothesis regarding CSF hydrodynamics has been proposed [1, 2]. According to this new hypothesis, CSF is permanently produced and absorbed inside the whole CSF system, as a consequence of filtration and reabsorption of water volume through the capillary walls into the interstitial fluid (ISF) of the surrounding brain tissue [1–3]. Furthermore, recent evidence has revealed the existence of a brain-wide network of paravascular channels, termed the “glymphatic” pathway, along which a large proportion of subarachnoid CSF recirculates through the brain parenchyma, facilitating the clearance of interstitial solutes, including amyloid-β (Aβ), from the brain [1, 4]. Although these concepts are also challenged and further studies are needed to substantiate their functional significance [1], one implication is that glymphatic pathway dysfunction may contribute to the deficient Aβ clearance in the pre-clinical stages of Alzheimer’s disease (AD) [5]. AD is thought to be caused by an imbalance between production and clearance of Aβ, leading to Aβ accumulation in the brain [6]. The new insights into the physiology of CSF circulation could also open new possibilities in the understanding of other neurodegenerative diseases in which the mis-accumulation of neurotoxic depositions contributes to disease development [1, 4]. In this paper, we review several lines of evidence showing that glaucoma may be an attractive candidate for such a disease. Interestingly, MRI-based imaging now provides a unique opportunity for investigating the role of the glymphatic system in glaucoma. If confirmed, this could lead to a new understanding of the pathogenesis of glaucoma.

## Discussion

### Glaucoma is a complex neurodegenerative disease

Glaucoma is one of the leading causes of irreversible blindness worldwide [7]. Primary open-angle glaucoma (POAG), the most common type, is a complex neurodegenerative disease characterized by slow progressive degeneration of retinal ganglion cells (RGCs) and their axons in the optic nerve, resulting in structural changes in the optic nerve head and corresponding visual field defects [7]. The optic nerve head is the likely site of initial injury and raised intraocular pressure (IOP) is considered a major risk factor for the development of POAG [7, 8]. However, elevated IOP is not present in all forms of POAG [7]. Indeed, in normal-tension glaucoma, IOP is not elevated and thus other risk factors must also be involved in the optic neuropathy of POAG [7].

**Figure 1:**
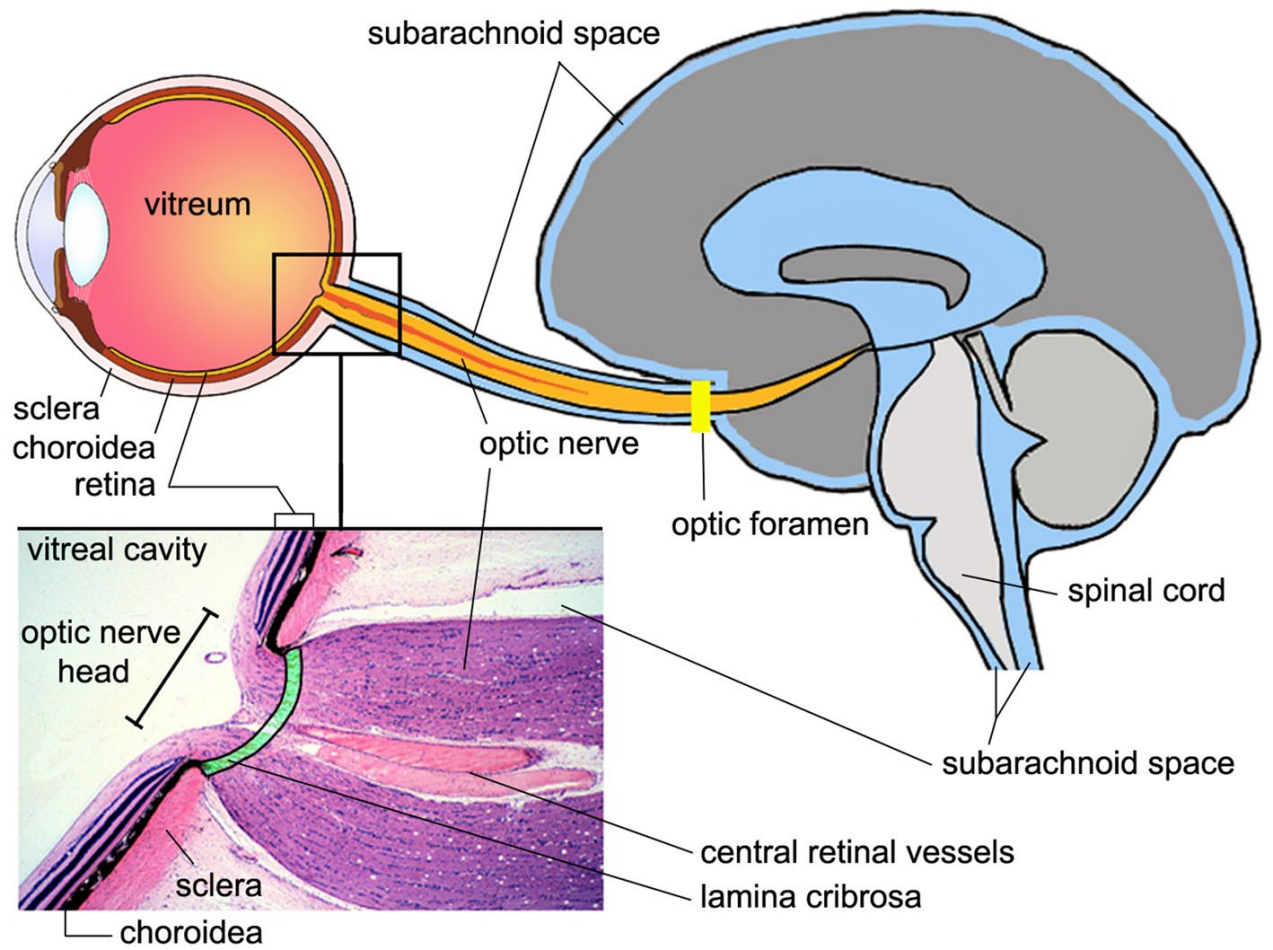
Section of eye, optic nerve head and brain, illustrating the subarachnoid space. The lamina cribrosa (*green*) is a sieve-like structure in the posterior part of the sclera and allows the passage of the retinal ganglion cell axons and the central retinal vessels. The optic nerve is surrounded by cerebrospinal fluid (*blue*) in the subarachnoid space. The enlarged histologic view is modified and reproduced with permission, Boston University Histology Learning System (Deborah W. Vaughan, PhD).

Growing evidence in the literature provides strong support for the concept that the CSF (both its pressure and composition) surrounding the optic nerve may have fundamental significance in the pathogenesis of glaucoma [9–12]. The optic nerve, a white matter tract of the central nervous system (CNS), is ensheathed in all three meningeal layers and surrounded by CSF in the SAS with a pressure equivalent to intracranial pressure (ICP) [10, 13]. Therefore, in addition to IOP, the optic nerve is exposed to the ICP [10]. The lamina cribrosa, the perforated region of the sclera through which the nerve fibers of the optic nerve pass as they exit the eye, separates these two pressurized regions [10]. The structures of the eye considered in this Commentary are illustrated in Figure 1.

### Accumulation of Aβ may be involved in RGC death in glaucoma

Considerable evidence indicates that Aβ may be implicated in the development of RGC apoptosis in glaucoma [14–17], suggesting a possible link with AD. Previous findings showed that there is IOP-sensitive increase in Aβ in glaucoma [14–17]. McKinnon et al. [14] reported that rat RGCs subjected to chronic elevation of IOP exhibit caspase-3-mediated abnormal processing of β-amyloid precursor protein (APP) with increased expression of Aβ. This suggested a new hypothesis for RGC death in glaucoma involving chronic Aβ neurotoxicity, mimicking AD at the molecular level [15]. Activation of caspases and abnormal APP processing, which includes production of Aβ, are also important events in AD [14]. Guo et al. [16] provided further evidence that Aβ is a likely mediator of pressure-induced RGC death. In a rat model mimicking chronic ocular hypertension, the authors found that Aβ colocalized with apoptotic RGCs [16]. They also demonstrated in vivo that Aβ induced significant RGC apoptosis [16]. The authors further provided evidence that targeting Aβ and blocking its effects with combination therapy may represent an effective treatment strategy in glaucoma [16]. Recently, in a study using monkeys with experimental glaucoma, Ito et al. [17] found time-dependent expressions and localization of Aβ in the retina as well as in the optic nerve head after chronic IOP elevation. It is interesting to note that a number of studies have similarly reported increased retinal Aβ in both AD transgenic mice and in human postmortem retinas of AD patients [18, 19].

### Decreased Aβ and increased tau in vitreous fluid and CSF

Studies consistently report decreased levels of Aβ (1–42) and increased levels of tau in CSF from AD patients in comparison with healthy subjects [20, 21]. Among AD patients, the consistent finding of low concentrations of Aβ (1–42) in CSF compared with those of age-matched controls is thought to be due to increased aggregation, fibril and plaque formation, with decreased clearance of these peptides from the CNS [22]. To test the idea that Aβ (1–42) and tau contribute to the development of glaucoma, Yoneda et al. [21] measured Aβ (1–42) and tau concentrations in vitreous fluid samples from eyes from patients with glaucoma. The authors found significantly decreased vitreous levels of Aβ (1–42) (consistent with Aβ deposition in the retina) and significantly increased vitreous levels of tau in patients with glaucoma in comparison with the levels in a control group [16, 21]. Their findings suggested that the neurodegenerative processes in glaucoma might share, at least in part, a common mechanism with AD [21]. It is also interesting to note that in a recent report, Nucci et al. [23] described a glaucoma patient with medically controlled IOP who experienced disease progression concomitantly with the onset of mild cognitive impairment and positivity for CSF markers of AD (decreased Aβ and elevated levels of total and phosphorylated tau). The authors suggested the possibility that altered CSF circulatory dynamics in this case reduced neurotoxin clearance along optic nerves in the SAS and that deposits/aggregates of tau and/or other toxic molecules may have contributed to the glaucoma progression.

### Flow of fluids in the anterior part of the optic nerve

With regard to the viewpoint presented here, it is interesting to note that previous studies investigating the flow of fluids in the anterior part of the optic nerve seem to confirm that there is at least some level of exchange between the ISF of the optic nerve and the surrounding CSF. These studies also demonstrated that the fluids from the vitreous body and the optic nerve move from opposite directions and converge at the optic nerve head [24, 25]. Interestingly, as noted above, in glaucoma, the optic nerve head is recognized as the likely site of initial injury [8].

In these investigations, electron microscopic studies and a variety of tracer substances were used [24]. The probable sources of ISF in the anterior part of the optic nerve were found to be capillaries in the nerve itself, peripapillary choroid, vitreous, CSF and possibly axoplasm in the local axons [24]. First of all, several studies found that there is a backward bulk flow of fluid from the vitreous into the optic nerve head [24–26]. Rodriguez-Peralta [25] investigated the subject in rabbits, cats, dogs, guinea pigs, and rhesus monkeys by injecting diaminoacridine dyes into the vitreous. The groups of animals receiving the tracer intravitreally showed that these compounds moved promptly peripherally toward the retina and notably toward the optic nerve head [25]. Bright fluorescence was found in the deeper layers of the retina and particularly in the optic nerve head, anterior to the lamina cribrosa [25]. The latter was the area of the highest concentrations of diaminoacridines [25]. Peyman and Apple [26] injected horseradish peroxidase into the vitreous of squirrel monkeys. They found that the tracer permeated readily into the optic nerve head and diffused freely through intercellular spaces of astrocytes, along collagen septa of the nerve and perivascular spaces [26]. There was no diffusion into capillaries of the nerve [26]. Hayreh [24] studied this backward flow from the vitreous along the optic nerve in rhesus monkeys by injecting tritiated leucine into the vitreous. The RGCs and the inner part of the retina had been first infarcted so that no axoplasmic transport was present [24]. Thus, the tracer seen in the optic nerve head represented a direct flow from the vitreous into the nerve, with no axoplasmic transport [24]. These studies revealed a heavy accumulation of the tracer in the glial cells in the anterior part of the prelaminar region, less accumulation in the posterior part of the prelaminar region, much less in the lamina cribrosa, and none or minimal accumulation in the retrolaminar optic nerve [24]. In addition to this backward bulk flow of fluid from the vitreous into the optic nerve head, several studies established that there is a flow of fluid from the SAS of the optic nerve into the optic nerve and optic nerve head [24, 25, 27, 28]. Rodriguez-Peralta [25] injected diaminoacridine dyes in the cisterna magna of rabbits, cats, dogs, guinea pigs, and rhesus monkeys. After 1 h, the dye was heavily concentrated in the SAS of the optic nerve and had diffused into the optic nerve and optic nerve head [25]. It was not found in the retina or vitreous body [25]. Tsukahara and Yamashita [27] injected horseradish peroxidase into the lateral ventricles of mice. 15 and 45 min later, the tracer was found in the SAS of the optic nerve [27]. It also penetrated freely into the optic nerve, and could be traced along the intercellular space between glial cells and optic nerve fibers and connective tissue septa, extending as far forward as the prelaminar region [27]. In his studies on rhesus monkeys, Hayreh [28] injected sodium fluorescein into the cisterna magna. On fluorescence photography of the optic disc in the living animal, usually within half an hour after the injection of the dye, the disc developed progressively increasing intense fluorescence and in 1.5–2 h it even started to diffuse into the vitreous [28]. Histological examination showed fluorescence of these tissues of the optic nerve and optic nerve head except in the lumen of vessels [28].

### Glymphatic pathway for CSF-ISF exchange in the optic nerve?

**Figure 2:**
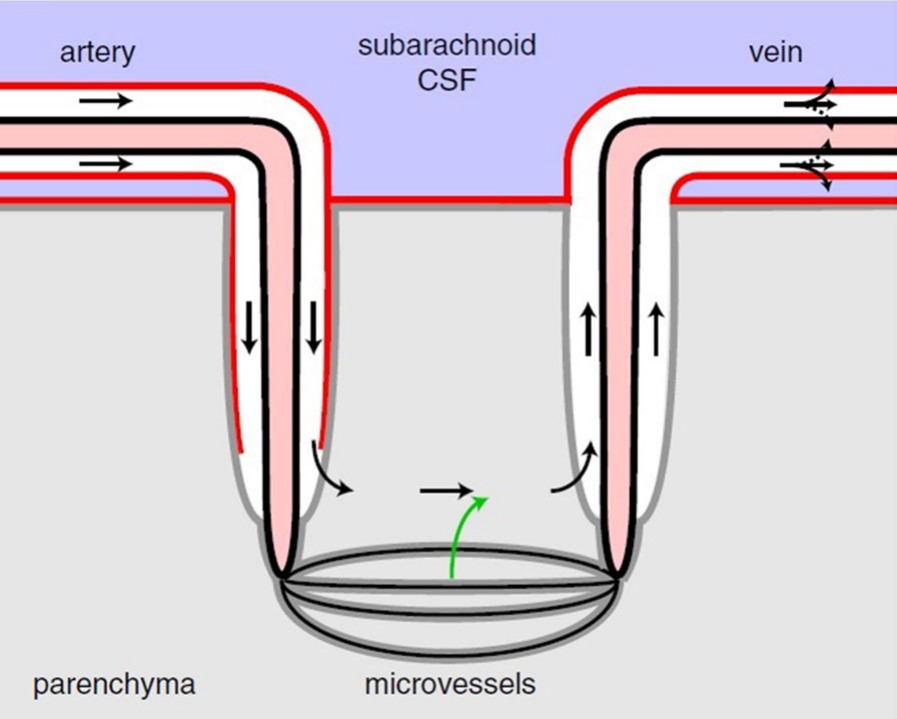
Schematic depiction of the glymphatic pathway in the brain. In this brain-wide pathway, cerebrospinal fluid from the subarachnoid space rapidly enters the brain along paravascular channels surrounding penetrating arteries and exchanges with brain interstitial fluid (ISF). ISF and parenchymal solutes (e.g., Aβ) are cleared from the brain along paravenous routes. The *green arrow* has been added as a reminder that fluid secreted by the blood–brain barrier contributes to the fluid in the parenchyma. Reproduced with permission from Hladky and Barrand [29], their Figure 9a (the glymphatic proposal).

Taken together, the above findings at least suggest some level of exchange between the ISF of the optic nerve and the surrounding CSF. Compared to the brain size, the optic nerve is a relatively small structure. In such small structures, simple diffusion of even large solutes may be sufficiently rapid to allow clearance (Iliff JJ, personal communication, 2013). However, apart from simple diffusion or conventional CSF bulk flow, the question is whether the optic nerve also uses paravascular pathways to facilitate more rapid exchange. Figure 2 is a schematic depiction of the glymphatic pathway in the brain. As an extension of the brain (Figure 1), the eye displays remarkable similarities to the brain in terms of anatomy and functionality [13]. Therefore, it would be very interesting to investigate whether a similar glymphatic pathway for CSF-ISF exchange exists in the optic nerve. However, even if this is not the case, there may be an interconnection between the glymphatic pathway of the brain and the SAS of the optic nerve, which in turn may facilitate efficient CSF-ISF exchange in the optic nerve, allowing clearance of interstitial solutes, including Aβ. Exploring this possibility could shed new light on the pathogenesis of glaucoma, and introduce a new look at the disease. Indeed, if confirmed, one might expect that a dysfunctional glymphatic system of the brain could ultimately result in reduced neurotoxin clearance in the optic nerve and lead to glaucomatous neurodegeneration. The observation of a dysfunctional glymphatic system in patients with glaucoma would also provide support for the hypothesis recently proposed by our group [7] that CSF circulatory dysfunction may play a contributory role in the pathogenesis of glaucomatous damage. Our viewpoint argues that glaucoma, just like AD, may occur when there is an imbalance between production and clearance of neurotoxins, including Aβ [30]. Our hypothesis is that the dominant alteration determines whether glaucoma manifests as normal-tension glaucoma or as high-tension glaucoma. In normal-tension glaucoma, abnormal clearance of Aβ from the optic nerve may predominate as a result of glymphatic pathway dysfunction. In high-tension glaucoma, IOP-induced Aβ generation may predominate and even mild impairment of glymphatic pathway function may result in glaucomatous optic nerve damage.

Interestingly, a growing body of evidence indicates that ICP is lower in patients with POAG when compared with nonglaucomatous control subjects and additionally, is lower in the normal-tension versus the high-tension form of POAG [9–11]. A decreased ICP can be the consequence of decreased CSF production or reduced resistance to CSF outflow. Indeed, the ICP is built up by the equilibrium between the production and outflow of CSF [7]. In this context, it is important to note that a recent study found that ICP decreases significantly and steadily after age 50 [31]. This parallels the rise in prevalence of glaucoma with increasing age [31]. There is no reported evidence that CSF outflow resistance decreases with age, rather most studies report CSF outflow resistance increases [31, 32]. However, there is evidence that the CSF production decreases with age [31, 33]. Therefore, the lower ICP reported in POAG patients could be an indicator of decreased CSF production and turnover [7]. Given that ICP is lower in patients with normal-tension glaucoma compared to patients with high-tension glaucoma, these changes in CSF circulatory physiology may be even more pronounced in the normal-tension compared to the high-tension form of POAG.

Also interestingly, the present viewpoint raises the possibility of explaining the clinical overlap of AD and glaucoma. Several studies have found an increased prevalence of glaucoma among patients with AD [34, 35]. In a nursing home-based study in Germany, Bayer et al. [34] studied 112 patients with AD and 116 control subjects. The prevalence of POAG was reported to be 25.9% in patients with AD and 5.2% in the control group. Glaucomatous visual field loss and/or optic disc cupping were the criteria for the diagnosis of glaucoma. In a Japanese study, Tamura et al. [35] found a prevalence of POAG of 23.8% among 172 patients with AD, which was significantly higher than the 9.9% among 176 control subjects. This higher rate of glaucoma among AD patients in combination with the multiple intriguing similarities between these two diseases has raised the question of whether AD and glaucoma may share a common underlying mechanism [36]. Glymphatic pathway dysfunction as a potential mechanistic link between AD and glaucoma is an attractive hypothesis since this could explain the coincidence of the two disorders.

Obviously, future studies are needed to elucidate the potential role of the glymphatic system in glaucoma. Interestingly, a recent study showed that the glymphatic system can be evaluated in the live rat brain using contrast-enhanced MRI following intracisternal gadolinium-based contrast infusion [37]. However, intracisternal infusions are rarely used in a clinical setting due to their high risk for developing iatrogenic complications, including traumatic tissue injury [38]. In contrast, the lumbar intrathecal route of administration is now commonly used in CT-myelography, anesthesia, pain management and chemotherapy [38]. Importantly, a recent pre-clinical study reported that perivascular CSF-ISF exchange within the rat brain can be evaluated following infusion of CSF tracer via a lumbar, in addition to an intracisternal, route [38]. The authors concluded that lumbar intrathecal contrast delivery is a clinically useful approach that could be used in conjunction with dynamic contrast-enhanced MRI nuclear imaging to assess glymphatic pathway function in humans [38]. This now provides a unique opportunity to evaluate whether suppression of the glymphatic system and thus CSF circulatory failure contributes to the development of glaucoma.

## Conclusion

Novel insights from molecular and cellular biology as well as neuroimaging research indicate that CSF physiology is much more complex than previously believed. The glymphatic system is a recently defined brain-wide paravascular pathway for CSF-ISF exchange that facilitates efficient clearance of interstitial solutes, including Aβ, from the brain. A newly developed theory proposes that failure of this clearance system underlies increased Aβ brain accumulation in AD. In this paper, we have reviewed several lines of evidence suggesting that the glymphatic system may also have potential clinical relevance for the understanding of glaucoma. It should be stressed that for the moment at least, the present hypothesis remains unproven. Future studies are needed to determine whether the glymphatic system may play a pathogenic role in the development of glaucoma. As a clinically acceptable MRI-based approach to evaluate glymphatic pathway function in humans has recently been developed, a unique opportunity now exists to investigate whether suppression of the glymphatic system and thus CSF circulatory dysfunction contributes to the development of glaucoma.

## Abbreviations

Aβ: Amyloid-β
AD: Alzheimer’s disease
APP: β-amyloid precursor protein
CNS: central nervous system
CSF: cerebrospinal fluid
ICP: intracranial pressure
IOP: intraocular pressure
ISF: interstitial fluid
POAG: primary open-angle glaucoma
RGC: retinal ganglion cell
SAS: subarachnoid space

## References

[1] Brinker T, Stopa E, Morrison J, Klinge P (2014). A new look at cerebrospinal fluid circulation. Fluids Barriers CNS.

[2] Oresković D, Klarica M (2010). The formation of cerebrospinal fluid: nearly a hundred years of interpretations and misinterpretations. Brain Res Rev.

[3] Igarashi H, Tsujita M, Kwee IL, Nakada T (2014). Water influx into cerebrospinal fluid is primarily controlled by aquaporin-4, not by aquaporin-1: 17O JJVCPE MRI study in knockout mice. NeuroReport.

[4] Iliff JJ, Wang M, Liao Y, Plogg BA, Peng W, Gundersen GA (2012). A paravascular pathway facilitates CSF flow through the brain parenchyma and the clearance of interstitial solutes, including amyloid β. Sci Transl Med..

[5] Yang L, Kress BT, Weber HJ, Thiyagarajan M, Wang B, Deane R (2013). Evaluating glymphatic pathway function utilizing clinically relevant intrathecal infusion of CSF tracer. J Transl Med.

[6] Mawuenyega KG, Sigurdson W, Ovod V, Munsell L, Kasten T, Morris JC (2010). Decreased clearance of CNS beta-amyloid in Alzheimer’s disease. Science.

[7] Wostyn P, De Groot V, Van Dam D, Audenaert K, De Deyn PP (2013). Senescent changes in cerebrospinal fluid circulatory physiology and their role in the pathogenesis of normal-tension glaucoma. Am J Ophthalmol.

[8] Johnson EC, Jia L, Cepurna WO, Doser TA, Morrison JC (2007). Global changes in optic nerve head gene expression after exposure to elevated intraocular pressure in a rat glaucoma model. Invest Ophthalmol Vis Sci.

[9] Berdahl JP, Allingham RR, Johnson DH (2008). Cerebrospinal fluid pressure is decreased in primary open-angle glaucoma. Ophthalmology.

[10] Berdahl JP, Fautsch MP, Stinnett SS, Allingham RR (2008). Intracranial pressure in primary open angle glaucoma, normal tension glaucoma, and ocular hypertension: a case- control study. Invest Ophthalmol Vis Sci.

[11] Ren R, Jonas JB, Tian G, Zhen Y, Ma K, Li S (2010). Cerebrospinal fluid pressure in glaucoma: a prospective study. Ophthalmology.

[12] Killer HE, Miller NR, Flammer J, Meyer P, Weinreb RN, Remonda L (2012). Cerebrospinal fluid exchange in the optic nerve in normal-tension glaucoma. Br J Ophthalmol.

[13] London A, Benhar I, Schwartz M (2013). The retina as a window to the brain-from eye research to CNS disorders. Nat Rev Neurol.

[14] McKinnon SJ, Lehman DM, Kerrigan-Baumrind LA, Merges CA, Pease ME, Kerrigan DF (2002). Caspase activation and amyloid precursor protein cleavage in rat ocular hypertension. Invest Ophthalmol Vis Sci.

[15] McKinnon SJ (2003). Glaucoma: ocular Alzheimer’s disease?. Front Biosci..

[16] Guo L, Salt TE, Luong V, Wood N, Cheung W, Maass A (2007). Targeting amyloid-beta in glaucoma treatment. Proc Natl Acad Sci USA.

[17] Ito Y, Shimazawa M, Tsuruma K, Mayama C, Ishii K, Onoe H (2012). Induction of amyloid-β (1-42) in the retina and optic nerve head of chronic ocular hypertensive monkeys. Mol Vis.

[18] Liu B, Rasool S, Yang Z, Glabe CG, Schreiber SS, Ge J (2009). Amyloid-peptide vaccinations reduce {beta}-amyloid plaques but exacerbate vascular deposition and inflammation in the retina of Alzheimer’s transgenic mice. Am J Pathol.

[19] Koronyo-Hamaoui M, Koronyo Y, Ljubimov AV, Miller CA, Ko MK, Black KL (2011). Identification of amyloid plaques in retinas from Alzheimer’s patients and noninvasive in vivo optical imaging of retinal plaques in a mouse model. Neuroimage.

[20] Engelborghs S, De Vreese K, Van de Casteele T, Vanderstichele H, Van Everbroeck B, Cras P (2008). Diagnostic performance of a CSF-biomarker panel in autopsy-confirmed dementia. Neurobiol Aging.

[21] Yoneda S, Hara H, Hirata A, Fukushima M, Inomata Y, Tanihara H (2005). Vitreous fluid levels of beta-amyloid((1-42)) and tau in patients with retinal diseases. Jpn J Ophthalmol.

[22] Silverberg GD, Mayo M, Saul T, Carvalho J, McGuire D (2004). Novel ventriculo-peritoneal shunt in Alzheimer’s disease cerebrospinal fluid biomarkers. Expert Rev Neurother.

[23] Nucci C, Martucci A, Martorana A, Sancesario GM, Cerulli L (2011). Glaucoma progression associated with altered cerebral spinal fluid levels of amyloid beta and tau proteins. Clin Experiment Ophthalmol.

[24] Hayreh SS (1978). Fluids in the anterior part of the optic nerve in health and disease. Surv Ophthalmol.

[25] Rodriguez-Peralta LA (1966). Hematic and fluid barriers in the optic nerve. J Comp Neurol.

[26] Peyman GA, Apple D (1972). Peroxidase diffusion processes in the optic nerve. Arch Ophthalmol.

[27] Tsukahara I, Yamashita H (1975). An electron microscopic study on the blood-optic nerve and fluid-optic nerve barrier. Albrecht Von Graefes Arch Klin Exp Ophthalmol.

[28] Hayreh SS (1977). Optic disc edema in raised intracranial pressure. V. Pathogenesis. Arch Ophthalmol.

[29] Hladky SB, Barrand MA (2014). Mechanisms of fluid movement into, through and out of the brain: evaluation of the evidence. Fluids Barriers CNS.

[30] Wostyn P, De Groot V, Van Dam D, Audenaert K, Killer HE, De Deyn PP (2014). Glaucoma considered as an imbalance between production and clearance of neurotoxins. Invest Ophthalmol Vis Sci.

[31] Fleischman D, Berdahl JP, Zaydlarova J, Stinnett S, Fautsch MP, Allingham RR (2012). Cerebrospinal fluid pressure decreases with older age. PLoS One.

[32] Albeck MJ, Skak C, Nielsen PR, Olsen KS, Børgesen SE, Gjerris F (1998). Age dependency of resistance to cerebrospinal fluid outflow. J Neurosurg.

[33] May C, Kaye JA, Atack JR, Schapiro MB, Friedland RP, Rapoport SI (1990). Cerebrospinal fluid production is reduced in healthy aging. Neurology.

[34] Bayer AU, Ferrari F, Erb C (2002). High occurrence rate of glaucoma among patients with Alzheimer’s disease. Eur Neurol.

[35] Tamura H, Kawakami H, Kanamoto T, Kato T, Yokoyama T, Sasaki K (2006). High frequency of open-angle glaucoma in Japanese patients with Alzheimer’s disease. J Neurol Sci.

[36] Bizrah M, Guo L, Cordeiro MF (2011). Glaucoma and Alzheimer’s disease in the elderly. Aging Health.

[37] Iliff JJ, Lee H, Yu M, Feng T, Logan J, Nedergaard M (2013). Brain-wide pathway for waste clearance captured by contrast-enhanced MRI. J Clin Invest.

[38] Yang L, Kress BT, Weber HJ, Thiyagarajan M, Wang B, Deane R (2013). Evaluating glymphatic pathway function utilizing clinically relevant intrathecal infusion of CSF tracer. J Transl Med.

